# The association of wrist circumference with hypertension in northeastern Chinese residents in comparison with other anthropometric obesity indices

**DOI:** 10.7717/peerj.7599

**Published:** 2019-08-28

**Authors:** Yongfang Li, Yuyan Liu, Jing He, Ping Ma, Luyang Yu, Guifan Sun

**Affiliations:** 1 Research Center of Environmental and Non-communicable Disease, School of Public Health, China Medical University, Shenyang, Liaoning, China; 2 Department of Clinical Epidemiology, The Fourth Affiliated Hospital of China Medical University, Shenyang, Liaoning, China; 3 Department of Non-Communicable Disease, Shenhe Center for Disease Control and Prevention, Shenyang, Liaoning, China

**Keywords:** Hypertension, Obesity, Wrist circumference, Blood pressure

## Abstract

**Background:**

Wrist circumference (WrC) is an easily obtained measure in estimating the body frame and regional fat distribution, and has increasingly used as an obesity index. The aim of our study is to estimate the association of WrC with elevated blood pressure (BP) among northeastern Chinese community-dwelling residents, and compare the strength of this association to other anthropometric obesity indices.

**Methods:**

A total of 2,331 adult participants (761 male participants, and 1,570 female participants) were included. WrC and other five generally used obesity indices, including body mass index (BMI), waist circumference (WC), waist-to-hip ratio (WHR), waist-to-height ratio (WHtR) and neck circumference (NC) were measured. Hypertension was defined as systolic blood pressure (SBP)/diastolic blood pressure (DBP) ≥140/90 mmHg or anti-hypertensive medication use. Multivariable linear and logistic regression models were performed to identify associations of BP and hypertension with per standard deviation (SD) increase of obesity indices. Areas under receiver operative characteristic curves (AUC) were calculated to compare the predicting capacity of WrC and other obesity indices on hypertension.

**Results:**

All of the six obesity indices were positively associated with both SBP and DBP after adjustment for age and gender (*P*-values of associations of SBP with obesity indices were 0.043 for WrC, and <0.001 for other five indices; *P*-values of associations of DBP with obesity indices were 0.011 for WrC, 0.031 for WHR, and <0.001 for other four indices), while the association between SBP and WrC showed no statistically significant after further adjusted for life-style and metabolic risk factors (*P*-value was 0.062). The increases of both SBP and DBP per SD increase of BMI were the largest. The positive associations of five obesity indices but WHR with hypertension were observed after adjustment for all risk factors (*P*-values were 0.024 for WrC, 0.064 for WHR and <0.001 for other four indices). However, the odd ratios (OR) of WrC was the smallest, while BMI was the largest. Consistently, the AUC of BMI was the largest and statistically larger than that observed for WrC (*P*-value <0.001).

**Conclusions:**

WrC was associated with hypertension among northeastern Chinese populations. However, the association was not stronger than the other generally used indices, particularly BMI.

## Introduction

Hypertension as a global health threat can increase the risk of cardiovascular diseases (CVD). Many lifestyle and metabolic risk factors can result in elevated blood pressure (BP) and hypertension, among which the association between hypertension and obesity is always highlighted (Kotsis et al., 2010; Browning, Hsieh & Ashwell, 2010; Lemieux et al., 2001). Anthropometric obesity indices including body mass index (BMI), waist circumference (WC), waist-to-hip ratio (WHR), waist-to-height ratio (WHtR) and neck circumference (NC) have been widely used to examine the obesity status, and studies from various populations have revealed the association between hypertension and these indices (Zhao et al., 2017; Ononamadu et al., 2017; Lee et al., 2015; Yang et al., 2010; Fan et al., 2017).

In addition to above generally used indices, wrist circumference (WrC) emerged as a novel anthropometric index in recent years, which is an easily obtained measure in estimating the body frame and regional fat distribution (Obirikorang, Obirikorang & Acheampong, 2018; Mueller et al., 2013; Komurcu, Kilic & Anlar, 2014; Zampetti et al., 2018a). Published findings showed that WrC was positively associated with several CVD risk factors, especially among adolescent populations (Zampetti et al., 2018b; Kajale et al., 2014; Kelishadi et al., 2017), while findings from adults are inconsistent (Hajsadeghi et al., 2016; Watkins et al., 2015; Kalantari & Khalili, 2017; Derakhshan et al., 2016). Additionally, no study showed if WrC was stronger associated with BP or hypertension compared to other anthropometric obesity indices.

The prevalence of hypertension in China is very high and has regional differences, with higher rate among northeastern Chinese residents than whom living in the south part (Yang et al., 2014; WHO, 2000). Although previous studies have shown generally used obesity indices (eg., BMI, WC, et al.,) was positively associated with hypertension in the specific populations, the association of WrC with BP and hypertension remained unknown. Therefore, in this study we are aiming to: (1) estimate the association of WrC with BP; and (2) compare the strength of this association to the other generally used anthropometric obesity indices among northeastern Chinese community-dwelling residents.

**Figure 1:**
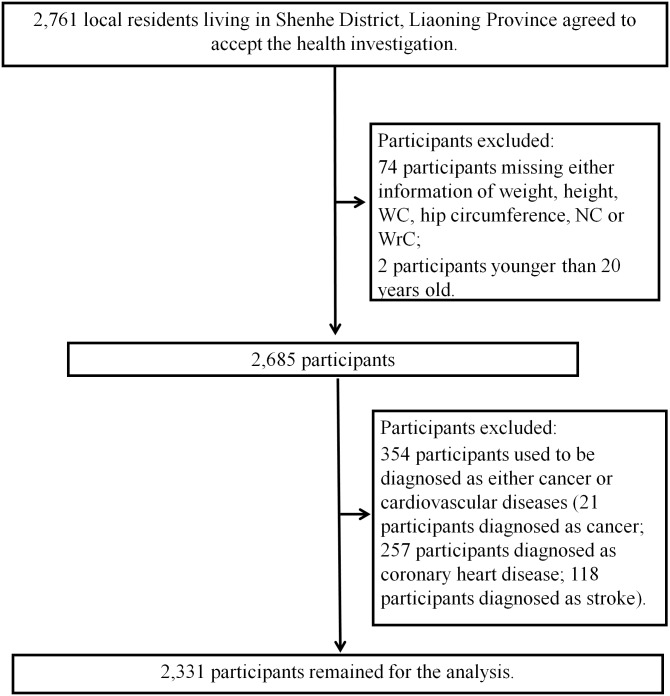
Flow chart for the present study.

## Materials & Methods

### Study design and participants

This is a cross-sectional study based on urban community-dwelling residents in Shenhe district, Shenyang City, Liaoning Province, China. Details of the methods of enrollment have been reported previously (Fan et al., 2017; Yang et al., 2017). In brief, in the first stage, all 14 blocks of Shenhe district were selected and eight residential communities were randomly selected from each of the blocks. In the second stage, systematic sampling was conducted to select 25 households in each residential community. In the third stage, within each household, residents older than 18 years old who lived in the district for at least 2 years were randomly selected without replacement. Finally, 2,761 local residents were recruited to finish the questionnaires and accepted the relevant physical examinations and biochemical tests. Among 2,761 participants, those who did not meet the inclusion criteria in this study were excluded as following: 74 participants missing either information of weight, height, WC, hip circumference, NC or WrC; 2 participants younger than 20 years old; and 354 participants who used to be diagnosed as either cancer or CVD (Fig. 1). As a result, 2,331 participants (761 male and 1,570 female participants) were remained for final analyses. Since all participants were randomly selected from communities without the consideration of gender, the total numbers of males and females were different.

To confirm the sample size involved in final analysis is enough or not, we calculate the minimum sample size required according to the following formula: $n={Z}_{1-\alpha /2}^{2}\times p(1-p)/{d}^{2}$ (Charan & Biswas, 2013). In this formula, *Z*_1−*α*∕2_ is standard normal variate (at 5% type I error (*P*-value <0.05), *Z*_1−*α*∕2_ is 1.96), *p* is the expected proportion in population based on previous studies, and d is the absolute error or precision (we decided *d* as 0.05 in our study). As a previous survey from northern Chinese population showed, the prevalence of hypertension was 48.9% and 30.8% in male and female participants, respectively (Sun et al., 2017). Therefore, the calculated minimum sample size for the current study were as following: *n* (male) = 1.96^2^ × 0.489 (1–0.489)/0.05^2^ = 384; *n* (female) = 1.96^2^ × 0.308 (1–0.308)/0.05^2^ = 328. The final sample size we selected for analysis was large enough compared to the number we calculated.

This study was in accordance with the World Medical Association Declaration of Helsinki-Ethical Principles for Medical Research Involving Human Subjects and was approved by the Ethics Committee of China Medical University (No: CMU62073024; July 15th, 2008). A written informed consent form was obtained from each participant after they were informed of the objectives and benefits of this study.

### Obesity indices

In this study, body weight, height and other circumferences were all measured twice using a vertical weight scale and a metric scale with standardized protocols, and to the nearest 0.1 kilogram (kg) and 0.1 centimeter (cm), respectively. All participants were asked to accept measuring of body weight and height wearing light clothes without shoes. BMI was then calculated as weight (kg) divided by the square of the height (m). WC was measured at the level of the midpoint between the lower rib margin and the iliac crest, and hip circumference was measured at the level of maximal protrusion of the gluteal muscles. WHR was calculated as WC (cm) divided by the hip circumference (cm). WHtR was calculated as WC (cm) divided by height (cm). NC was measured at the level of the laryngeal prominence while participants were asked to be in the standing position with the head held erect and eyes facing forward. WrC was measured when participants were asked to hold their arm on a flat surface, and the superior border of the tape measure was placed just distal to the prominences of radial and ulnar bones.

### Measuring of BP and the definition of hypertension

BP was measured using a standard mercury-column sphygmomanometer after 15 min of rest in the sitting position based on the standardized procedural guideline (Kirkendall et al., 1981). Systolic blood pressure (SBP) and diastolic blood pressure (DBP) were determined by the first and the fifth Korotkoff sounds. We measured BP on both right and left arm firstly, and the arm with higher blood pressure would be selected to obtain three consecutive measurements with a time interval of at least two minutes (Pickering et al., 2005). Then, the average on the selected arm would be recorded as the final BP. Before measuring, participants were also need to be asked for not doing following behaviors, including drinking alcohol, tea or coffee, smoking, or taking any exercise for at least 30 min before measuring BP. In this study, hypertension was defined as systolic blood pressure (SBP)/diastolic blood pressure (DBP) ≥140/90 mmHg or anti-hypertensive medication use (Pickering et al., 2005).

### Other risk factor measurements

All participants were asked for permission to collect a blood sample after an overnight fast of longer than 8 h. The concentrations of total cholesterol (TC), triglycerides (TG), high density lipid cholesterol (HDL-C), and fasting glucose were examined using Mindray Autoanalyzer (BS 380 type; Mindray Ltd.; Shenzhen, China) in local community health service centers. All assays were performed according to the manufacturer’s instructions. In addition, self-administered questionnaires were also used to obtain information on demographic characteristics, medical history, medication use, smoking habits, and other pertinent factors. Trained staff members confirmed the reported information with each participant. In this study, smoking status was categorized as current smokers (smoking during the last one year or quit smoking for less than six months), past smokers (quit smoking for more than six months), and never. Drinking status were categorized as current drinkers (at least twice per week for male and once per week for female), past drinkers (quit drinking for more than six months), and never.

### Statistical analysis

We have tested distributions of all continuous variables using Shapiro–Wilk test, and found that all these variables followed normal distributions (*p*-value >0.05). Therefore, we showed values of all continuous variables in mean and standard deviation (SD). Categorical variables were presented as percentages. Student t test and chi-square test were respectively used to estimate the difference of continuous and categorical variables between male and female. Six obesity indices were estimated in this study as following: BMI, WC, WHR, WHtR, NC and WrC. All six anthropometric obesity indices were standardized by being divided by their SD, respectively, so that crude and adjusted slopes per SD increase of each obesity index could be compared with each other. Multivariable linear regression models were used to estimate the crude and adjusted associations of BP (SBP and DBP) with per SD increase of each obesity index among participants without any anti-hypertensive medication use. Odds ratio (OR) and 95% confidence interval (95%CI) were used to estimate the risk of hypertension per SD increase of each obesity index using multivariable logistic regression models. In this study, we used three models in above-mentioned linear and logistic regressions: Model 1 (unadjusted), Model 2 (adjusted for age and gender), Model 3 (adjusted for smoking, alcohol drinking, fasting glucose, TG, TC and HDL-C in addition to Model 2). The ability of predicting hypertension using each obesity index together with other risk factors (confounders in Model 3) was examined by receiver operative characteristic (ROC) curves. We thereafter compared areas under ROC curves (AUC) of other 5 obesity indices to that of the index whose OR was largest in multivariable logistic regression model. The Bonferroni adjustment for multiple comparisons was used to adjust *p*-values. The subgroup analysis for gender was also performed, and *p*-values of interactions by gender on associations of obesity indices with BP and hypertension were estimated. All statistical analyses were performed by SAS 9.4 software (SAS Institute, Inc., Cary, North Carolina), and *p*-value <0.05 was considered as statistical significance.

## Results

### Baseline characteristics

Baseline characteristics of 2,331 participants are showed in the Table 1. As it is shown, male participants were generally older than female participants. Except WHtR, the other five obesity indices (i.e., BMI, WC, WHR, NC, WrC) in male subjects were larger than that in female subjects (*P*-values were 0.293 for WHtR, and <0.001 for other five indices). Also, male participants showed higher levels of SBP and DBP than female participants (*p*-values were <0.001). The prevalence of hypertension among male participants (42.4%) was also found higher than that in females (35.5%) (*P*-value was 0.001). Additionally, male participants showed dramatically higher rates of current smokers (24.3%) and drinkers (51.5%) (*p*-values were <0.001).

**Table 1:** Characteristics of Participants from Shenhe District, Liaoning Province, China (2,331 participants, aged 20 to 94 years old, examined in 2015).

	Male participants (*n* = 761)	Female participants (*n* = 1,570)	*P*-value
Age (years old)	58.9 ± 11.2^*^	57.7 ± 11.6	0.013
Weight (kg)	73.3 ± 10.8^*^	62.0 ± 9.0	<0.001
Height (cm)	172.1 ± 5.1^*^	160.2 ± 4.7	<0.001
BMI (kg/m^2^)	24.7 ± 3.4^*^	24.2 ± 3.3	<0.001
WC (cm)	89.4 ± 9.0^*^	82.8 ± 8.3	<0.001
WHtR	0.5 ± 0.1	0.5 ± 0.1	0.293
Hip circumference (cm)	99.7 ± 8.8^*^	95.8 ± 8.7	<0.001
WHR	0.9 ± 0.1^*^	0.9 ± 0.1	<0.001
NC (cm)	37.5 ± 4.6^*^	34.4 ± 5.1	<0.001
WrC (cm)	19.2 ± 4.4^*^	17.0 ± 4.0	<0.001
SBP (mmHg)	131.9 ± 12.1^*^	127.4 ± 14.5	<0.001
DBP (mmHg)	81.5 ± 8.3^*^	78.0 ± 8.5	<0.001
Fasting glucose (mmol/L)	5.9 ± 1.8	5.7 ± 2.8	0.103
TG (mmol/L)	2.2 ± 6.4	1.8 ± 1.1	0.066
TC (mmol/L)	5.0 ± 1.0^*^	5.3 ± 1.2	<0.001
HDL-C (mmol/L)	1.4 ± 0.4^*^	1.5 ± 0.5	<0.001
Hypertension (%)	42.4^*^	35.5	0.001
Antihypertensive medication (%)	27.5^*^	23.1	0.020
Smoking (%)			<0.001
Current	24.3^*^	1.5	
Past	16.0	2.0	
Never	59.7	96.5	
Drinking (%)			<0.001
Current	51.5^*^	14.3	
Past	2.6	0.8	
Never	45.9	84.9	

**Notes.**

Values are presented as mean ± SD, or %.

Abbreviations BMI body mass index WC waist circumference WHR waist-to-hip ratio WHtR waist-to-height ratio NC neck circumference WrC wrist circumference SBP systolic blood pressure DBP diastolic blood pressure TG triglycerides TC total cholesterol HDL-C high density lipid cholesterol

* *p* < 0.05, statistically significant.

**Table 2:** Crude and adjusted associations of blood pressure with per standard deviation increase of obesity indices (1,760 participants without anti-hypertensive medication, aged 20 to 94 years old, examined in 2015).

	Model 1			Model 2			Model 3		
	BP	95% CI	*P*-value	BP	95% CI	*P*-value	BP	95% CI	*P*-value
**SBP**									
BMI	2.73^*^	2.13, 3.33	<0.001	2.52^*^	1.96, 3.08	<0.001	2.45^*^	1.89, 3.02	<0.001
WC	3.22^*^	2.64, 3.80	<0.001	2.49^*^	1.89, 3.08	<0.001	2.39^*^	1.79, 3.00	<0.001
WHR	1.73^*^	1.14, 2.32	<0.001	1.12^*^	0.55, 1.69	<0.001	1.13^*^	0.55, 1.71	<0.001
WHtR	2.72^*^	2.16, 3.29	<0.001	2.10^*^	1.56, 2.64	<0.001	2.01^*^	1.47, 2.55	<0.001
NC	2.53^*^	1.89, 3.16	<0.001	1.49^*^	0.85, 2.12	<0.001	1.35^*^	0.71, 2.00	<0.001
WrC	1.40^*^	0.76, 2.03	<0.001	0.64^*^	0.02, 1.25	0.043	0.59	−0.03, 1.20	0.062
**DBP**									
BMI	1.73^*^	1.36, 2.10	<0.001	1.59^*^	1.23, 1.95	<0.001	1.53^*^	1.17, 1.89	<0.001
WC	1.81^*^	1.44, 2.17	<0.001	1.33^*^	0.94, 1.71	<0.001	1.23^*^	0.84, 1.61	<0.001
WHR	0.80^*^	0.43, 1.17	<0.001	0.41^*^	0.04, 0.78	0.031	0.41^*^	0.04, 0.78	0.031
WHtR	1.36^*^	1.00, 1.71	<0.001	1.10^*^	0.75, 1.45	<0.001	1.02^*^	0.68, 1.37	<0.001
NC	1.46^*^	1.07, 1.86	<0.001	0.88^*^	0.48, 1.29	<0.001	0.68^*^	0.27, 1.09	0.001
WrC	0.95^*^	0.56, 1.35	<0.001	0.51^*^	0.12, 0.91	0.011	0.49^*^	0.10, 0.88	0.014

**Notes.**

Model 1: unadjusted; Model 2: adjusted for age and gender; Model 3: adjusted for smoking, alcohol drinking, fasting glucose, TG, TC, and HDL-C in addition to Model 2.

Abbreviations BP blood pressure SBP systolic blood pressure DBP diastolic blood pressure BMI body mass index WC waist circumference WHR waist-to-hip ratio WHtR waist-to-height ratio NC neck circumference WrC wrist circumference

* *p* < 0.05, statistically significant.

### The associations of WrC and the other obesity indices with BP

**Figure 2:**
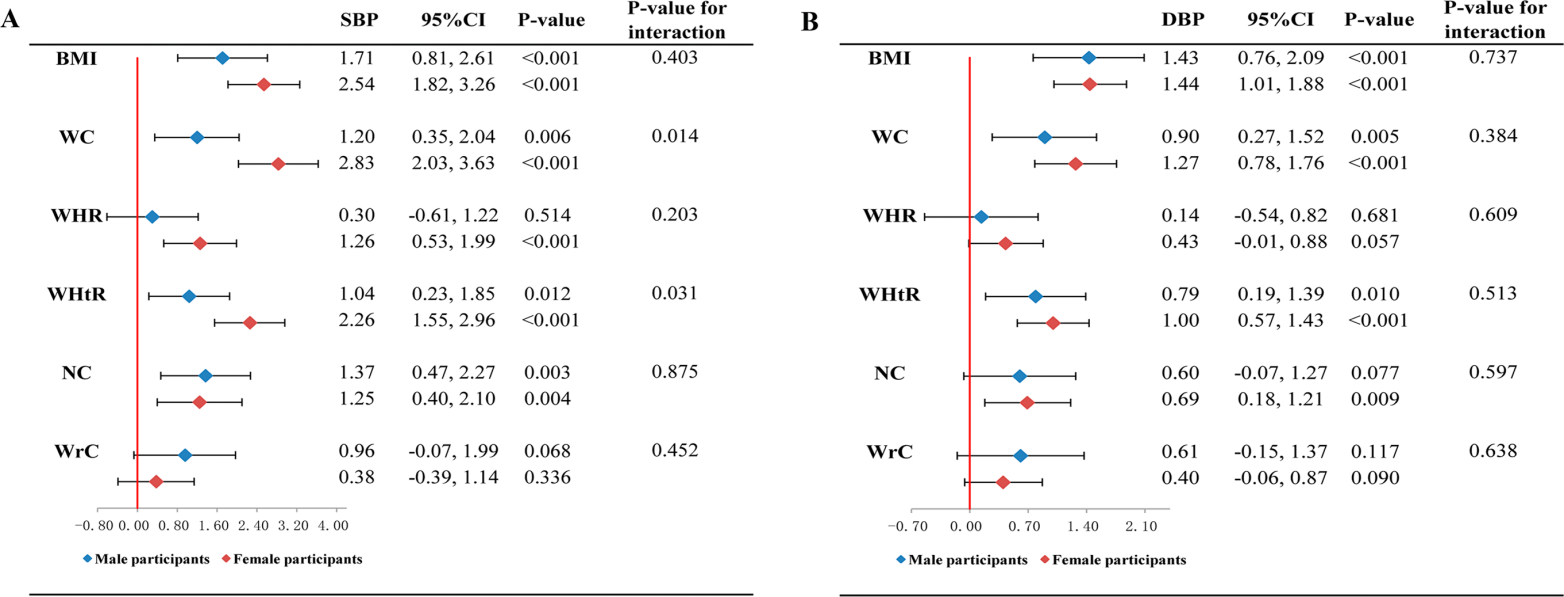
Association of systolic blood pressure (A) and diastolic blood pressure (B) with obesity indices classified by gender (1,760 participants without anti-hypertensive medication, examined in 2015). The linear regression model was adjusted for age, smoking, alcohol drinking, fasting glucose, TG, TC, and HDL-C (male participants, *n* = 552; female participants, *n* = 1, 208) **Abbreviations:** BMI, body mass index; WC, waist circumference; WHR, waist-to-hip ratio; WHtR, waist-to-height ratio; NC, neck circumference; WrC, wrist circumference.

Table 2 shows the crude (Model 1) and adjusted (Model 2, 3) associations of SBP and DBP with per SD increase of each obesity indices. In the Model 1, WrC and other 5 obesity indices were all found positively associated with both SBP and DBP (*p*-values were <0.001). These positive associations remained present in the Model 2, which further adjusted for age and gender (*p*-values of associations of SBP with obesity indices were 0.043 for WrC, and <0.001 for other five indices; *p*-values of associations of DBP with obesity indices were 0.011 for WrC, 0.031 for WHR, and <0.001 for other four indices). However, in the Model 3, the association between WrC and SBP was not statistically significant (*P*-value was 0.062). In contrast, the other five obesity indices remained the positive associations with both SBP and DBP (*p*-values of association of SBP with five obesity indices but WrC were <0.001; *p*-values of associations of DBP with obesity indices were 0.031 for WHR, and <0.001 for other four indices but NC, whose *P*-value was 0.001). Furthermore, comparison of the increases of BP per SD of each obesity indices showed that BMI was the largest, with 2.52 mmHg for SBP and 1.59 mmHg for DBP in Model 2 and 2.45 mmHg for SBP and 1.53 mmHg for DBP in Model 3. In gender stratified analyses, no associations of WrC with SBP and DBP were found in either male or female participants after adjustment for all risk factors (Fig. 2) (*p*-values for SBP were 0.068 and 0.336 in male and female; *p*-values for DBP were 0.117 and 0.090 in male and female). On the contrary, the positive associations of BMI, WC and WHtR with SBP and DBP were found, respectively in both male and female participants even after consideration of all risk factors (Fig. 2) (*p*-values of association of SBP with BMI were <0.001 in both male and female; *p*-values of association of SBP with WC were 0.006 and <0.001 in male and female; *p*-values of association of SBP with WHtR were 0.012 and <0.001 in male and female; *p*-values of association of DBP with BMI were <0.001 in both male and female; *p*-values of association of DBP with WC were 0.005 and <0.001 in male and female; *p*-values of association of DBP with WHtR were 0.010 and <0.001 in male and female). Besides, we also found stronger associations of both WC and WHtR with SBP in female than that in male (*p*-values for interactions by gender were 0.014 and 0.031 for WC and WHtR, respectively) (Fig. 2A).

### The associations of WrC and the other obesity indices with hypertension

**Figure 3:**
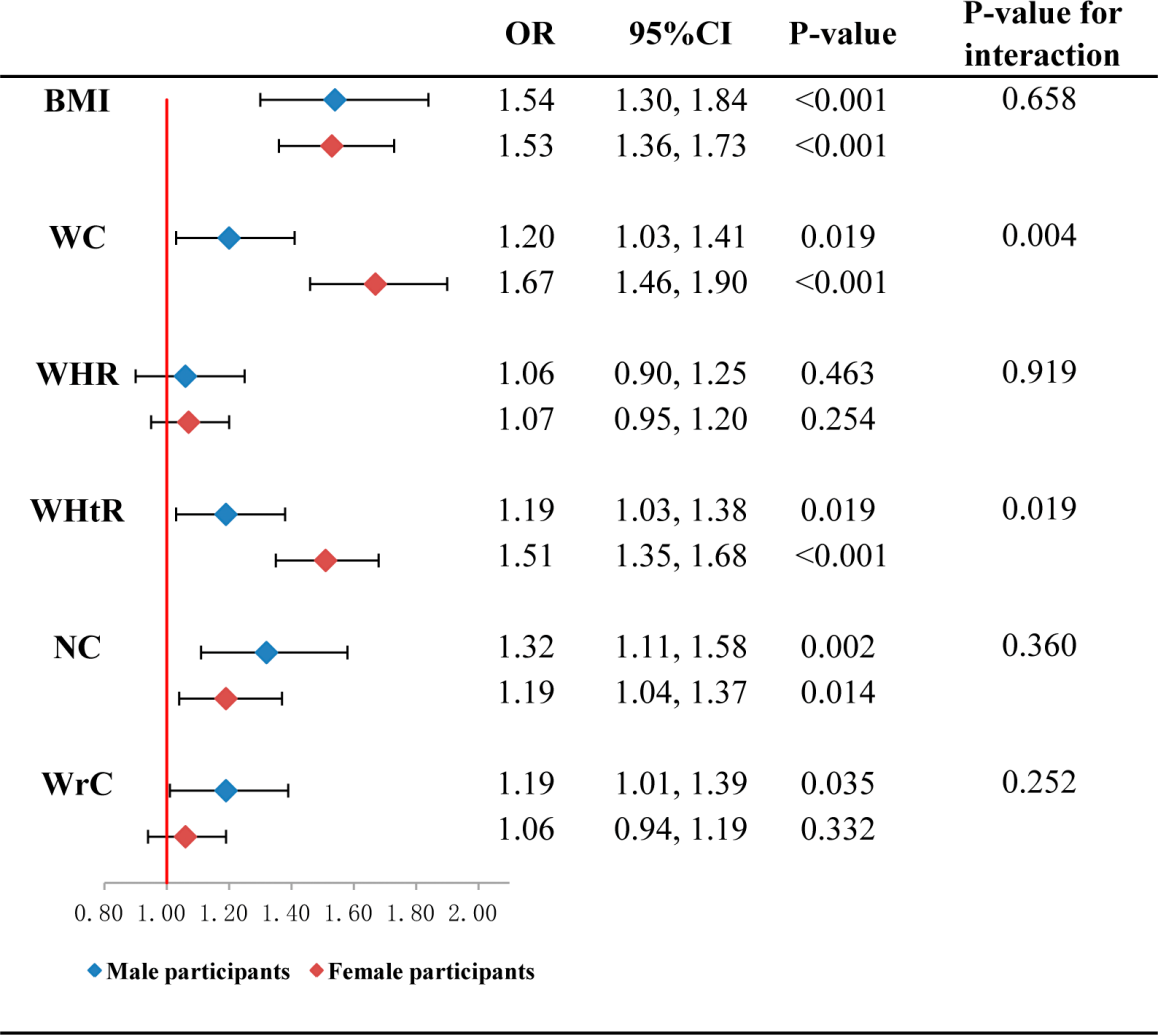
Association of hypertension with per standard deviation of obesity indices classified by gender (2,331 participants aged 20 to 94 years old, examined in 2015). The logistic regression model was adjusted for age, smoking, alcohol drinking, fasting glucose, TG, TC, and HDL-C (male participants, *n* = 761; female participants, *n* = 1,570) **Abbreviations:** OR, odds ratio; BMI, body mass index; WC, waist circumference; WHR, waist-to-hip ratio; WHtR, waist-to-height ratio; NC, neck circumference; WrC, wrist circumference.

The associations of the prevalence of hypertension and obesity indices are shown in the Table 3. The findings suggested positive associations of five obesity indices but WHR with hypertension even after adjustments for all risk factors in Model 3 (*P*-values were 0.024 for WrC, 0.064 for WHR and <0.001 for other four indices). However, OR values of WrC kept the smallest (OR was 1.16, 95 CI% [1.07–1.27] in Model 1; OR was 1.11, 95 CI% [1.02–1.21] in Model 2; and OR was 1.11, 95 CI% [1.01–1.21] in Model 3, respectively) compared with the other five indices. The association of BMI with hypertension was strongest after adjustments for risk factors (OR was 1.59, 95 CI% [1.44–1.75] in Model 2; and OR was 1.57, 95 CI% [1.42–1.73] in Model 3). Gender stratified analyses showed that WrC was only associated with hypertension in male after adjustment for all risk factors (*P*-value was 0.035), while no interaction by gender was found (Fig. 3) (*P*-value for interaction was 0.252). In contrast, BMI, WC and WHtR were found positively associated with hypertension in both male and female participants after adjusting for all risk factors (Fig. 3) (*P*-values for BMI were <0.001 in both male and female; *p*-values for WC were 0.019 and <0.001 in male and female; *p*-values for WHtR were 0.019 and <0.001 in male and female). Additionally, the associations of WC and WHtR with hypertension in female were found strongly higher than that in male (*p*-values for interaction by gender were 0.004 and 0.019 for WC and WHtR, respectively) (Fig. 3).

**Table 3:** Crude and adjusted Associations of hypertension with per standard deviation increase of obesity indices (2,331 participants, aged 20 to 94 years old, examined in 2015).

	Model 1			Model 2			Model 3		
	OR	95%CI	*P*-value	OR	95%CI	*P*-value	OR	95%CI	*P*-value
BMI	1.53^*^	1.40, 1.68	<0.001	1.59^*^	1.44, 1.75	<0.001	1.57^*^	1.42, 1.73	<0.001
WC	1.54^*^	1.41, 1.68	<0.001	1.52^*^	1.38, 1.68	<0.001	1.49^*^	1.35, 1.65	<0.001
WHR	1.16^*^	1.06, 1.26	<0.001	1.11^*^	1.01, 1.22	0.029	1.09	1.00, 1.20	0.064
WHtR	1.50^*^	1.38, 1.63	<0.001	1.44^*^	1.32, 1.57	<0.001	1.41^*^	1.29, 1.54	<0.001
NC	1.34^*^	1.21, 1.48	<0.001	1.25^*^	1.12, 1.39	<0.001	1.25^*^	1.12, 1.40	<0.001
WrC	1.16^*^	1.07, 1.27	<0.001	1.11^*^	1.02, 1.21	0.023	1.11^*^	1.01, 1.21	0.024

**Notes.**

Model 1: unadjusted; Model 2: adjusted for age and gender; Model 3: adjusted for smoking, alcohol drinking, fasting glucose, TG, TC, and HDL-C in addition to Model 2.

Abbreviations OR odds ratio BMI body mass index WC waist circumference WHR waist-to-hip ratio WHtR waist-to-height ratio NC neck circumference WrC wrist circumference

* *p* < 0.05, statistically significant.

Furthermore, we compared the AUC of each obesity indices in predicting hypertension under the control of risk factors. As Table 4 shows, the AUC of BMI was the largest (0.709) among the 6 obesity indices. Compared to BMI, the AUC of WHR (AUC = 0.674), NC (AUC = 0.681) and WrC (AUC = 0.674) were found significantly lower (adjusted *p*-values were <0.001). Similar results were also suggested in female participants (Table 4).

**Table 4:** Areas under receiver operating characteristic curves for predicting hypertension by obesity indices and other factors (2,331 participants, aged 20 to 94 years old, examined in 2015). AUC of all five obesity indices except BMI were compared with that of BMI; other factors include age, gender, smoking, alcohol drinking, fasting glucose, TG, TC, and HDL-C; *p*-values were adjusted using Bonferroni adjusment.

	AUC	95%CI	*P*-value	Adjusted *p*-value
BMI+other factors	0.709	0.680, 0.730	ref.	ref.
WC + other factors	0.699	0.677, 0.720	0.101	1.000
WHR + other factors	0.674^*^	0.653, 0.696	<0.001	<0.001
WHtR + other factors	0.697	0.675, 0.718	0.045	0.675
NC + other factors	0.681^*^	0.659, 0.718	<0.001	<0.001
WrC + other factors	0.674^*^	0.652, 0.695	<0.001	<0.001
Male Participants (*n* = 761)				
BMI + other factors	0.671	0.633, 0.709	ref.	ref.
WC + other factors	0.639	0.600, 0.678	0.006	0.090
WHR + other factors	0.634	0.595, 0.673	0.004	0.060
WHtR + other factors	0.638	0.599, 0.677	0.004	0.060
NC + other factors	0.646	0.607, 0.685	0.072	1.000
WrC + other factors	0.643	0.604, 0.682	0.039	0.585
Female Participants (*n* = 1,570)				
BMI + other factors	0.726	0.701, 0.761	ref.	ref.
WC + other factors	0.728	0.702, 0.753	0.746	1.000
WHR + other factors	0.699^*^	0.673, 0.725	<0.001	<0.001
WHtR + other factors	0.725	0.699, 0.750	0.870	1.000
NC + other factors	0.702^*^	0.676, 0.728	<0.001	0.006
WrC + other factors	0.697^*^	0.671, 0.724	<0.001	<0.001

**Notes.**

Abbreviations AUC area under curve BMI body mass index WC waist circumference WHR waist-to-hip ratio WHtR waist-to-height ratio NC neck circumference WrC wrist circumference

* *p* < 0.05, statistically significant.

## Discussion

In summary, we found that WrC was positively associated with hypertension but only associated with DBP after adjustments for all confounders. The positive association of BP with WrC is not stronger than that observed in other generally used obesity indices. Consistently, the capacity of WrC in predicting hypertension was also found lower than BMI, WC and WHtR, which recognized as the most frequently used obesity indices.

WrC has attracted much attention in recent years, and became more and more popular in both clinical and research settings (Namazi et al., 2018a). Previous studies found a regulator of osteoblastic proliferation, differentiation and bone matrix apposition, named as insulin-like growth factor 1 (IGF-1), which showed 40% similarity in amino acid structure with insulin (Fulzele et al., 2010; Kinjo, Setoguchi & Solomon, 2007). Therefore, hyperinsulinemia was considered to be correlated with the bone growth (Ferron et al., 2010), and WrC as a good surrogate assessing the cross-sectional area of bone then could be used to estimate the risks of insulin resistance, which was closely correlated with elevated BP. Meanwhile, some studies reported that individuals who are obese or overweight showed greater bone mass (Leonard et al., 2004; Clark, Ness & Tobias, 2006), which additionally implied that as a good measure of body frame and regional fat distribution (Obirikorang, Obirikorang & Acheampong, 2018; Mueller et al., 2013; Komurcu, Kilic & Anlar, 2014; Zampetti et al., 2018a), WrC could be used as an obesity index. Based on above knowledge and the strong positive association of obesity with CVD risk, several studies focusing on the associations of WrC with elevated BP as well as other metabolic abnormalities emerged (Kelishadi et al., 2017; Namazi et al., 2018a; Jahangiri Noudeh et al., 2013; Mohebi et al., 2014), while no finding from Chinese populations was published. For example, Kelishadi et al. found that WrC was positively associated with BP and the metabolic syndrome, and inversely associated with HDL-C among 3,843 children and adolescents living in Iran (Kelishadi et al., 2017). A cohort study (the mean follow-up of 8.8 years) from Iran suggested the positive associations of WrC with both metabolic syndrome (hazard ratio was 1.32 in male, and 1.40 in female) and diabetes (hazard ratio was 1.17 in male, and 1.31 in female) (Jahangiri Noudeh et al., 2013). Another 10-year-follow-up study from 3,642 asymptomatic Iran female participants (≥30 years old) showed that after adjustment for other conventional risk factors, WrC was still positively associated with hypertension (hazard ratio: 1.15; 95%CI [1.06–1.25]) (Mohebi et al., 2014). In contrast with previous findings, our study firstly showed that WrC is positive associated with hypertension after adjusting for confounding factors among northeastern Chinese population, particularly in male. The findings were in line with previous reports (Kelishadi et al., 2017; Namazi et al., 2018a; Clark, Ness & Tobias, 2006; Jahangiri Noudeh et al., 2013; Mohebi et al., 2014; Capizzi et al., 2011).

Additionally, the advantages of WrC measurements compared to other anthropometric obesity indices are continually emphasized. In contrast to WC, WrC was independent of respiration and stomach fullness (Namazi et al., 2018b; Ross et al., 2008). Also, no special assessment tools are required for WrC measurement, which led it easier to apply in health examination (Jahangiri Noudeh et al., 2013). However, no studies have compared the strengths of associations of WrC with metabolic abnormalities to other anthropometric obesity indices, using the standardized coefficients of each index. If the association of WrC was no stronger than other indices, it means that WrC do not need be measured additionally, and consumptions of the human power and the training for examiners can be saved. According to our current results, the positive association of WrC and hypertension is not stronger than the other traditional obesity indices. Firstly, Table 2 indicated us that WrC is only positive associated with DBP and the increase of DBP per SD of WrC is smallest compared to other obesity indices. Secondly, Table 3 showed that the OR values of WrC and hypertension was also revealed to be smallest among the six obesity indices. Additionally, the AUC values comparison in Table 4 further demonstrated that the capacity of WrC in predicting hypertension is statistically lower than BMI. All of these findings consistently implied that the WrC did not show better performance compared to the other traditional obesity indices with regard to the association with hypertension. On the contrary, the three conventional obesity indices, BMI, WC and WHtR, showed more strongly association with hypertension, with BMI as the strongest one. And thus, it should be more recommended in detecting the risk of hypertension in our specific population.

Cautions should be paid when interpreting our findings. Firstly, as a cross-sectional study, the causality between obesity indices and hypertension cannot be proven well, longitudinal following-up study should be made further to verify the current results. Secondly, only local residents were recruited from a single area in this study. Therefore, conclusions from this study may be different in other populations with various life-styles. Thirdly, several life-style factors correlated with hypertension, such as dietary intakes and physical activity were not considered in our study. Hypertension is a multifactorial and multigenic disease and then further studies focusing on associations of hypertension with both genetic factors and life-style factors should be continued. One of strengths in our study was that we randomly selected a community-based sample with a broad age range, which increases the generalizability of our results to general Chinese population. Secondly, we included not only WrC, but also other generally used anthropometric obesity indices into analyses, and compared strengths of associations with hypertension. Such systematic comparisons have not been reported previously, especially in northeastern Chinese population. We supposed that the current findings might provide a reference in selecting the appropriate anthropometric obesity indices to estimate the risk of hypertension.

## Conclusions

In the current study, we showed that WrC was associated with hypertension among Chinese northeastern populations, especially in adult males. However, the association was not stronger than other generally used obesity indices, particularly BMI. Our study suggested that WrC, as a novel anthropometric obesity index, to some extent can be used as a surrogate to estimate the risk of hypertension, while the application value was limited. In contrast, other commonly used obesity indices showed much more well performances in detecting hypertension risk. Thus, additional measurement of WrC is not recommended at least with regard to the issue of hypertension in this specific population.

##  Supplemental Information

10.7717/peerj.7599/supp-1 Data S1 Raw data

10.7717/peerj.7599/supp-2 Supplemental Information 1 The codebook for the categorical raw data

10.7717/peerj.7599/supp-3 Supplemental Information 2 The SAS code used in this study

## Additional Information and Declarations

### Data Availability

The following information was supplied regarding data availability: The raw data, statistical code, and the codebook are available in the Supplemental Files.
