# Supplementary material for: The association of wrist circumference with hypertension in northeastern Chinese residents in comparison with other anthropometric obesity indices
Source: PeerJ. 2019 Aug 28;7:e7599. doi: 10.7717/peerj.7599 (PMC6717503; doi:10.7717/peerj.7599)
Supplement: Supplemental Information 1 [file peerj-07-7599-s002.docx]

Translation of Variables in Database:

“gender”: gender of participants

“1”=men;

“2”=women

“history1”, “history2”, “history3”, “history4”: disease history

blank=no disease;

“1”=hypertension;

“2”=coronary heart disease;

“3”=stroke;

“4”=diabetes;

“5”=cancer;

“6”=COPD;

“7”=other diseases;

“8”=dyslipdemia

“htnmed1”, “htnmed2”, “htnmed3”, “htnmed4”: medications of hypertension

“0”=no medication

“1”=yes

“diamed1”, “diamed2”, “diamed3”, “diamed4”: medications of diabetes

“0”=no medication

“1”=yes

“hlipmed1”, “hlipmed2”, “hlipmed3”, “hlipmed4”: medications of dyslipidemia

“0”=no medication

“1”=yes

“actsmok”: active smoking

“1”= >40 cigarettes per day

“2”= 20-40 cigarettes per day;

“3”= <20 cigarettes per day;

“4”= stop smoking for less than 3 months;

“5”= stop smoking for more than 3 months;

“6”= never smoke

“drinking”: alcohol drinking

“1”= current drinking;

“2”= past drinking;

“3”= never drinking
