# Supplementary material for: The association of wrist circumference with hypertension in northeastern Chinese residents in comparison with other anthropometric obesity indices
Source: PeerJ. 2019 Aug 28;7:e7599. doi: 10.7717/peerj.7599 (PMC6717503; doi:10.7717/peerj.7599)
Supplement: Supplemental Information 2 [file peerj-07-7599-s003.docx]

*sort the database;

libname yuyan "D:\goupi\useful database CMU";

proc import datafile="D:\goupi\useful database CMU\shenhe database 2761 for SAS1"

out=yuyan.shenhe2761

dbms=excel replace;

sheet="sheet1$";

run;

data analysis0;

set yuyan.shenhe2761;

if height=. then delete;

if weight=. then delete;

if waist=. then delete;

if wrist=. then delete;

if hip=. then delete;

if neck=. then delete;

bmi=weight/((height/100)**2);

whratio=waist/hip;

whtratio=waist/height;

if age lt 20 then delete;

if history1=5 or history2=5 or history3=5 or history4=5 then delete;

if history1=2 or history2=2 or history3=2 or history4=2 then delete;

if history1=3 or history2=3 or history3=3 or history4=3 then delete;

run;

*definitions of hypertension;

data hyperten; set analysis0;

if history1=1 or history2=1 or history3=1 or history4=1 then htn=1;

else if sbp ge 140 or dbp ge 90 then htn=1;

else if sbp=. or dbp=. then htn=.;

else htn=0;

run;

proc print ; where sbp=. or dbp=.;

var id sbp dbp history1 history2 history3 history4 htn;

run;

*+++++++++++++++++++++++++++++++++++++++++++++++++++++++;

*+++++++++++++++++++++++++++++++++++++++++++++++++++++++++++++++++++++++++++++++++++++++++++++++++++++++++;

proc freq;

tables htn0*gender;

run;

proc freq;

tables htn*gender;

run;

*baseline characteristics excluding extreme values;

data analysis1;

set hyperten;

if BMI lt 16 or BMI gt 50 then delete;

if waist lt 40 or waist gt 130 then delete;

if wrist lt 3 or wrist gt 90 then delete;

if hip lt 40 or hip gt 200 then delete;

if neck lt 15 or neck gt 150 then delete;

run;

proc tabulate;

class gender;

var age weight height hip bmi waist wrist neck whratio whtratio hcy sbp dbp glu tg tc hdl ldl;

table age weight height hip bmi waist wrist

neck whratio whtratio hcy sbp dbp glu tg tc hdl ldl, gender*(n mean std);

run;

proc tabulate;

var age bmi waist wrist neck whratio whtratio hcy sbp dbp glu tg tc hdl ldl;

table age bmi waist wrist neck whratio whtratio hcy sbp dbp glu tg tc hdl ldl,(n min p25 median p75 max mean std);

run;

proc freq;

tables gender*(actsmok drinking);

run;

proc freq;

tables htn*gender;

run;

*age classified;

data analysis9;

set analysis1;

if age lt 40 then agep=1;

else if age ge 40 and age lt 65 then agep=2; else agep=3;

if age lt 65 then ageg=1; else ageg=2;

run;

proc tabulate;

class gender;

var age bmi waist neck wrist whratio whtratio sbp dbp glu tg tc hdl;

table age bmi waist neck wrist whratio whtratio sbp dbp glu tg tc hdl, gender*(n p25 median p75 mean std);

run;

proc tabulate;

var age bmi waist neck wrist whratio whtratio sbp dbp glu tg tc hdl;

table age bmi waist neck wrist whratio whtratio sbp dbp glu tg tc hdl,(n mean std);

run;

proc univariate plot normal;

var age bmi waist neck wrist whratio whtratio sbp dbp glu tg tc hdl;

histogram;

run;

proc tabulate;

class agep;

var age bmi waist neck wrist whratio whtratio sbp dbp glu tg tc;

table age bmi waist neck wrist whratio whtratio sbp dbp glu tg tc, agep*(n mean);

run;

proc tabulate;

class ageg;

var age bmi waist neck wrist whratio whtratio sbp dbp glu tg tc;

table age bmi waist neck wrist whratio whtratio sbp dbp glu tg tc, ageg*(mean);

run;

proc corr spearman;

var bmi waist whratio whtratio wrist htn diabetes hlip;

partial age gender;

run;

proc ttest;

class gender;

var age weight height hip bmi waist neck whratio whtratio wrist sbp dbp glu tg tc hdl;

run;

proc freq;

tables (htn actsmok drinking)*gender/chisq;

run;

proc freq;

tables htn *gender/chisq;

run;

*logistic regression model;

*differences from p25 to p75 of obesity indices in both male and female groups are similar with that of general participants,

then values of general participants are used;

data analysis2;

set analysis9;

bmi_t=bmi/3.31;

waist_t=waist/9.08;

whratio_t=whratio/0.07;

whtratio_t=whtratio/0.05;

wrist_t=wrist/4.24;

neck_t=neck/5.13;

run;

proc logistic descending;

model htn=bmi_t/cl;

run;

proc logistic descending;

model htn=waist_t/cl;

run;

proc logistic descending;

model htn=whratio_t/cl;

run;

proc logistic descending;

model htn=whtratio_t/cl;

run;

proc logistic descending;

model htn=neck_t/cl;

run;

proc logistic descending;

model htn=wrist_t/cl;

run;

proc logistic descending;

model htn=bmi_t age gender/cl;

run;

proc logistic descending;

model htn=waist_t age gender/cl;

run;

proc logistic descending;

model htn=whratio_t age gender/cl;

run;

proc logistic descending;

model htn=whtratio_t age gender/cl;

run;

proc logistic descending;

model htn=neck_t age gender/cl;

run;

proc logistic descending;

model htn=wrist_t age gender/cl;

run;

proc logistic descending;

model htn=bmi_t age gender actsmok drinking glu tg tc hdl/cl;

run;

proc logistic descending;

model htn=waist_t age gender actsmok drinking glu tg tc hdl/cl;

run;

proc logistic descending;

model htn=whratio_t age gender actsmok drinking glu tg tc hdl/cl;

run;

proc logistic descending;

model htn=whtratio_t age gender actsmok drinking glu tg tc hdl/cl;

run;

proc logistic descending;

model htn=neck_t age gender actsmok drinking glu tg tc hdl/cl;

run;

proc logistic descending;

model htn=wrist_t age gender actsmok drinking glu tg tc hdl/cl;

run;

*gender interaction;

proc logistic descending;

class gender;

model htn=bmi_t*gender bmi_t age gender actsmok drinking glu tg tc hdl/cl;

run;

proc logistic descending;

class gender;

model htn=waist_t*gender waist_t age gender actsmok drinking glu tg tc hdl/cl;

run;

proc logistic descending;

class gender;

model htn=whratio_t*gender whratio_t age gender actsmok drinking glu tg tc hdl/cl;

run;

proc logistic descending;

class gender;

model htn=whtratio_t*gender whtratio_t age gender actsmok drinking glu tg tc hdl/cl;

run;

proc logistic descending;

class gender;

model htn=neck_t*gender neck_t age gender actsmok drinking glu tg tc hdl/cl;

run;

proc logistic descending;

class gender;

model htn=wrist_t*gender wrist_t age gender actsmok drinking glu tg tc hdl/cl;

run;

*there is no roc plot in following "proc logistic" codes;

ods graphics on;

proc logistic descending plots(only)=roc;

model htn = bmi waist wrist whratio whtratio neck age gender actsmok drinking glu tg tc hdl;

roc "bmi" bmi age gender actsmok drinking glu tg tc hdl;

roc "waist" waist age gender actsmok drinking glu tg tc hdl;

roc "wrist" wrist age gender actsmok drinking glu tg tc hdl;

roc "wh" whratio age gender actsmok drinking glu tg tc hdl;

roc "wht" whtratio age gender actsmok drinking glu tg tc hdl;

roc "neck" neck age gender actsmok drinking glu tg tc hdl;

roccontrast reference ("bmi")/estimate e;

run;

data pvalues;

input raw_p;

datalines;

0.101

0.0001

0.045

0.0001

0.0001

;

proc sort; by raw_p;

proc multtest pdata=pvalues bon holm fdr;

run;

ods graphics off;

*+++++++++++++++++++++++++++;

*+++++++++++++++++++++++++++++;

*+++++++++++++++++++++++++++++++;

*+++++++++++++++++++++++++++++++;

*+++++++++++++++++++++++++++;

*very important;

proc freq;

tables actsmok*htn/chisq trend;

run;

*++++++++++++++++++++++++++++++++++;

*+++++++++++++++++++++++++++++++++;

*+++++++++++++++++++++++++++++++++;

*++++++++++++++++++++++++++++;

*+++++++++++++++++++++++++++++;

*selecting ones who do not get antihypertensive medication among participants with hypertension;

data analysis3;

set analysis2;

if htnmed1=1 or htnmed2=1 or htnmed3=1 or htnmed4=1 then htnmed=1;

else htnmed=0;

run;

proc freq;

tables htnmed*htn;

run;

proc freq;

tables htnmed*gender/chisq;

run;

proc freq;

tables htnmed0*htn;

run;

*linear regression model blood pressure&obesity;

proc glm; where htnmed=0;

model sbp=bmi_t/solution clparm;

run;

proc glm; where htnmed=0;

model sbp=waist_t/solution clparm;

run;

proc glm; where htnmed=0;

model sbp=wrist_t/solution clparm;

run;

proc glm; where htnmed=0;

model sbp=whratio_t/solution clparm;

run;

proc glm; where htnmed=0;

model sbp=whtratio_t/solution clparm;

run;

proc glm; where htnmed=0;

model sbp=neck_t/solution clparm;

run;

proc glm; where htnmed=0;

model sbp=bmi_t age gender/solution clparm;

run;

proc glm; where htnmed=0;

model sbp=waist_t age gender/solution clparm;

run;

proc glm; where htnmed=0;

model sbp=wrist_t age gender/solution clparm;

run;

proc glm; where htnmed=0;

model sbp=whratio_t age gender/solution clparm;

run;

proc glm; where htnmed=0;

model sbp=whtratio_t age gender/solution clparm;

run;

proc glm; where htnmed=0;

model sbp=neck_t age gender/solution clparm;

run;

proc glm; where htnmed=0;

model sbp=bmi_t age gender actsmok drinking glu tg tc hdl/solution clparm;

run;

proc glm; where htnmed=0;

model sbp=waist_t age gender actsmok drinking glu tg tc hdl/solution clparm;

run;

proc glm; where htnmed=0;

model sbp=wrist_t age gender actsmok drinking glu tg tc hdl/solution clparm;

run;

proc glm; where htnmed=0;

model sbp=whratio_t age gender actsmok drinking glu tg tc hdl/solution clparm;

run;

proc glm; where htnmed=0;

model sbp=whtratio_t age gender actsmok drinking glu tg tc hdl/solution clparm;

run;

proc glm; where htnmed=0;

model sbp=neck_t age gender actsmok drinking glu tg tc hdl/solution clparm;

run;

*gender interaction;

proc glm; where htnmed=0;

class gender;

model sbp= bmi_t age gender bmi_t*gender actsmok drinking glu tg tc hdl/solution clparm;

run;

proc glm; where htnmed=0;

class gender;

model sbp= waist_t age gender waist_t*gender actsmok drinking glu tg tc hdl/solution clparm;

run;

proc glm; where htnmed=0;

class gender;

model sbp= wrist_t age gender wrist_t*gender actsmok drinking glu tg tc hdl/solution clparm;

run;

proc glm; where htnmed=0;

class gender;

model sbp= whratio_t age gender whratio_t*gender actsmok drinking glu tg tc hdl/solution clparm;

run;

proc glm; where htnmed=0;

class gender;

model sbp=whtratio_t age gender whtratio_t*gender actsmok drinking glu tg tc hdl/solution clparm;

run;

proc glm; where htnmed=0;

class gender;

model sbp=neck_t age gender neck_t*gender actsmok drinking glu tg tc hdl/solution clparm;

run;

*++++++++++++++++++++++++++++++++++++++++++++++++++++++++++++++++++++++++++++++++++++++;

proc glm; where htnmed=0;

model dbp=bmi_t/solution clparm;

run;

proc glm; where htnmed=0;

model dbp=waist_t/solution clparm;

run;

proc glm; where htnmed=0;

model dbp=wrist_t/solution clparm;

run;

proc glm; where htnmed=0;

model dbp=whratio_t/solution clparm;

run;

proc glm; where htnmed=0;

model dbp=whtratio_t/solution clparm;

run;

proc glm; where htnmed=0;

model dbp=neck_t/solution clparm;

run;

proc glm; where htnmed=0;

model dbp=bmi_t age gender/solution clparm;

run;

proc glm; where htnmed=0;

model dbp=waist_t age gender/solution clparm;

run;

proc glm; where htnmed=0;

model dbp=wrist_t age gender/solution clparm;

run;

proc glm; where htnmed=0;

model dbp=whratio_t age gender/solution clparm;

run;

proc glm; where htnmed=0;

model dbp=whtratio_t age gender/solution clparm;

run;

proc glm; where htnmed=0;

model dbp=neck_t age gender/solution clparm;

run;

proc glm; where htnmed=0;

model dbp=bmi_t age gender actsmok drinking glu tg tc hdl/solution clparm;

run;

proc glm; where htnmed=0;

model dbp=waist_t age gender actsmok drinking glu tg tc hdl/solution clparm;

run;

proc glm; where htnmed=0;

model dbp=wrist_t age gender actsmok drinking glu tg tc hdl/solution clparm;

run;

proc glm; where htnmed=0;

model dbp=whratio_t age gender actsmok drinking glu tg tc hdl/solution clparm;

run;

proc glm; where htnmed=0;

model dbp=whtratio_t age gender actsmok drinking glu tg tc hdl/solution clparm;

run;

proc glm; where htnmed=0;

model dbp=neck_t age gender actsmok drinking glu tg tc hdl/solution clparm;

run;

*gender interaction;

proc glm; where htnmed=0;

class gender;

model dbp= bmi_t age gender bmi_t*gender actsmok drinking glu tg tc hdl/solution clparm;

run;

proc glm; where htnmed=0;

class gender;

model dbp= waist_t age gender waist_t*gender actsmok drinking glu tg tc hdl/solution clparm;

run;

proc glm; where htnmed=0;

class gender;

model dbp= wrist_t age gender wrist_t*gender actsmok drinking glu tg tc hdl/solution clparm;

run;

proc glm; where htnmed=0;

class gender;

model dbp= whratio_t age gender whratio_t*gender actsmok drinking glu tg tc hdl/solution clparm;

run;

proc glm; where htnmed=0;

class gender;

model dbp=whtratio_t age gender whtratio_t*gender actsmok drinking glu tg tc hdl/solution clparm;

run;

proc glm; where htnmed=0;

class gender;

model dbp=neck_t age gender neck_t*gender actsmok drinking glu tg tc hdl/solution clparm;

run;

*++++++++++++++++++++++++++++++++++++++++++++++++++++++++++++++++++++++++++;

*++++++++++++++++++++++++++++++++++++++++++++++++++++++++++++++++++++++++++;

*++++++++++++++++++++++++++++++++++++++++++++++++++++++++++++++++++++++++++;

*male;

data analysis7;

set analysis3;

if gender=2 then delete;

run;

proc logistic descending;

model htn=bmi_t/cl;

run;

proc logistic descending;

model htn=waist_t/cl;

run;

proc logistic descending;

model htn=whratio_t/cl;

run;

proc logistic descending;

model htn=whtratio_t/cl;

run;

proc logistic descending;

model htn=neck_t/cl;

run;

proc logistic descending;

model htn=wrist_t/cl;

run;

proc logistic descending;

model htn=bmi_t age /cl;

run;

proc logistic descending;

model htn=waist age /cl;

run;

proc logistic descending;

model htn=whratio_t age /cl;

run;

proc logistic descending;

model htn=whtratio_t age /cl;

run;

proc logistic descending;

model htn=neck_t age /cl;

run;

proc logistic descending;

model htn=wrist_t age /cl;

run;

proc logistic descending;

model htn=bmi_t age actsmok drinking glu tg tc hdl/cl;

run;

proc logistic descending;

model htn=waist_t age actsmok drinking glu tg tc hdl/cl;

run;

proc logistic descending;

model htn=whratio_t age actsmok drinking glu tg tc hdl/cl;

run;

proc logistic descending;

model htn=whtratio_t age actsmok drinking glu tg tc hdl/cl;

run;

proc logistic descending;

model htn=neck_t age actsmok drinking glu tg tc hdl/cl;

run;

proc logistic descending;

model htn=wrist_t age actsmok drinking glu tg tc hdl/cl;

run;

*there is no roc plot in following "proc logistic" codes;

ods graphics on;

proc logistic descending plots(only)=roc;

model htn = bmi waist wrist whratio whtratio neck age actsmok drinking glu tg tc hdl;

roc "bmi" bmi age actsmok drinking glu tg tc hdl;

roc "waist" waist age actsmok drinking glu tg tc hdl;

roc "wrist" wrist age actsmok drinking glu tg tc hdl;

roc "wh" whratio age actsmok drinking glu tg tc hdl;

roc "wht" whtratio age actsmok drinking glu tg tc hdl;

roc "neck" neck age actsmok drinking glu tg tc hdl;

roccontrast reference ("bmi")/estimate e;

run;

data pvalues;

input raw_p;

datalines;

0.006

0.004

0.004

0.072

0.039

;

proc sort; by raw_p;

proc multtest pdata=pvalues bon holm fdr;

run;

*linear regression model blood pressure&obesity;

data analysis14;

set analysis7;

if htnmed1=1 or htnmed2=1 or htnmed3=1 or htnmed4=1 then htnmed=1;

else htnmed=0;

run;

proc glm; where htnmed=0;

model sbp=bmi_t/solution clparm;

run;

proc glm; where htnmed=0;

model sbp=waist_t/solution clparm;

run;

proc glm; where htnmed=0;

model sbp=wrist_t/solution clparm;

run;

proc glm; where htnmed=0;

model sbp=whratio_t/solution clparm;

run;

proc glm; where htnmed=0;

model sbp=whtratio_t/solution clparm;

run;

proc glm; where htnmed=0;

model sbp=neck_t/solution clparm;

run;

proc glm; where htnmed=0;

model sbp=bmi_t age /solution clparm;

run;

proc glm; where htnmed=0;

model sbp=waist_t age /solution clparm;

run;

proc glm; where htnmed=0;

model sbp=wrist_t age /solution clparm;

run;

proc glm; where htnmed=0;

model sbp=whratio_t age/solution clparm;

run;

proc glm; where htnmed=0;

model sbp=whtratio_t age/solution clparm;

run;

proc glm; where htnmed=0;

model sbp=neck_t age /solution clparm;

run;

proc glm; where htnmed=0;

model sbp=bmi_t age actsmok drinking glu tg tc hdl/solution clparm;

run;

proc glm; where htnmed=0;

model sbp=waist_t age actsmok drinking glu tg tc hdl/solution clparm;

run;

proc glm; where htnmed=0;

model sbp=wrist_t age actsmok drinking glu tg tc hdl/solution clparm;

run;

proc glm; where htnmed=0;

model sbp=whratio_t age actsmok drinking glu tg tc hdl/solution clparm;

run;

proc glm; where htnmed=0;

model sbp=whtratio_t age actsmok drinking glu tg tc hdl/solution clparm;

run;

proc glm; where htnmed=0;

model sbp=neck_t age actsmok drinking glu tg tc hdl/solution clparm;

run;

*++++++++++++++++++++++++++++++++++++++++++++++++++++++++++++++++++++++++++++++++++++++;

proc glm; where htnmed=0;

model dbp=bmi_t/solution clparm;

run;

proc glm; where htnmed=0;

model dbp=waist_t/solution clparm;

run;

proc glm; where htnmed=0;

model dbp=wrist_t/solution clparm;

run;

proc glm; where htnmed=0;

model dbp=whratio_t/solution clparm;

run;

proc glm; where htnmed=0;

model dbp=whtratio_t/solution clparm;

run;

proc glm; where htnmed=0;

model dbp=neck_t/solution clparm;

run;

proc glm; where htnmed=0;

model dbp=bmi_t age /solution clparm;

run;

proc glm; where htnmed=0;

model dbp=waist_t age /solution clparm;

run;

proc glm; where htnmed=0;

model dbp=wrist_t age /solution clparm;

run;

proc glm; where htnmed=0;

model dbp=whratio_t age /solution clparm;

run;

proc glm; where htnmed=0;

model dbp=whtratio_t age /solution clparm;

run;

proc glm; where htnmed=0;

model dbp=neck_t age /solution clparm;

run;

proc glm; where htnmed=0;

model dbp=bmi_t age actsmok drinking glu tg tc hdl/solution clparm;

run;

proc glm; where htnmed=0;

model dbp=waist_t age actsmok drinking glu tg tc hdl/solution clparm;

run;

proc glm; where htnmed=0;

model dbp=wrist_t age actsmok drinking glu tg tc hdl/solution clparm;

run;

proc glm; where htnmed=0;

model dbp=whratio_t age actsmok drinking glu tg tc hdl/solution clparm;

run;

proc glm; where htnmed=0;

model dbp=whtratio_t age actsmok drinking glu tg tc hdl/solution clparm;

run;

proc glm; where htnmed=0;

model dbp=neck_t age actsmok drinking glu tg tc hdl/solution clparm;

run;

*++++++++++++++++++++++++++++++++++++++++++++++++++++++++++++++++++++++++++;

*female;

data analysis8;

set analysis3;

if gender=1 then delete;

run;

proc logistic descending;

model htn=bmi_t/cl;

run;

proc logistic descending;

model htn=waist_t/cl;

run;

proc logistic descending;

model htn=whratio_t/cl;

run;

proc logistic descending;

model htn=whtratio_t/cl;

run;

proc logistic descending;

model htn=neck_t/cl;

run;

proc logistic descending;

model htn=wrist_t/cl;

run;

proc logistic descending;

model htn=bmi_t age /cl;

run;

proc logistic descending;

model htn=waist age/cl;

run;

proc logistic descending;

model htn=whratio_t age/cl;

run;

proc logistic descending;

model htn=whtratio_t age/cl;

run;

proc logistic descending;

model htn=neck_t age/cl;

run;

proc logistic descending;

model htn=wrist_t age /cl;

run;

proc logistic descending;

model htn=bmi_t age actsmok drinking glu tg tc hdl/cl;

run;

proc logistic descending;

model htn=waist_t age actsmok drinking glu tg tc hdl/cl;

run;

proc logistic descending;

model htn=whratio_t age actsmok drinking glu tg tc hdl/cl;

run;

proc logistic descending;

model htn=whtratio_t age actsmok drinking glu tg tc hdl/cl;

run;

proc logistic descending;

model htn=neck_t age actsmok drinking glu tg tc hdl/cl;

run;

proc logistic descending;

model htn=wrist_t age actsmok drinking glu tg tc hdl/cl;

run;

*there is no roc plot in following "proc logistic" codes;

ods graphics on;

proc logistic descending plots(only)=roc;

model htn = bmi waist wrist whratio whtratio neck age actsmok drinking glu tg tc hdl;

roc "bmi" bmi age actsmok drinking glu tg tc hdl;

roc "waist" waist age actsmok drinking glu tg tc hdl;

roc "wrist" wrist age actsmok drinking glu tg tc hdl;

roc "wh" whratio age actsmok drinking glu tg tc hdl;

roc "wht" whtratio age actsmok drinking glu tg tc hdl;

roc "neck" neck age actsmok drinking glu tg tc hdl;

roccontrast reference ("bmi")/estimate e;

run;

data pvalues;

input raw_p;

datalines;

0.746

0.0001

0.870

0.0005

0.0001

;

proc sort; by raw_p;

proc multtest pdata=pvalues bon holm fdr;

run;

*linear regression model blood pressure&obesity;

data analysis15;

set analysis8;

if htnmed1=1 or htnmed2=1 or htnmed3=1 or htnmed4=1 then htnmed=1;

else htnmed=0;

run;

proc glm; where htnmed=0;

model sbp=bmi_t/solution clparm;

run;

proc glm; where htnmed=0;

model sbp=waist_t/solution clparm;

run;

proc glm; where htnmed=0;

model sbp=wrist_t/solution clparm;

run;

proc glm; where htnmed=0;

model sbp=whratio_t/solution clparm;

run;

proc glm; where htnmed=0;

model sbp=whtratio_t/solution clparm;

run;

proc glm; where htnmed=0;

model sbp=neck_t/solution clparm;

run;

proc glm; where htnmed=0;

model sbp=bmi_t age /solution clparm;

run;

proc glm; where htnmed=0;

model sbp=waist_t age/solution clparm;

run;

proc glm; where htnmed=0;

model sbp=wrist_t age /solution clparm;

run;

proc glm; where htnmed=0;

model sbp=whratio_t age/solution clparm;

run;

proc glm; where htnmed=0;

model sbp=whtratio_t age /solution clparm;

run;

proc glm; where htnmed=0;

model sbp=neck_t age /solution clparm;

run;

proc glm; where htnmed=0;

model sbp=bmi_t age actsmok drinking glu tg tc hdl/solution clparm;

run;

proc glm; where htnmed=0;

model sbp=waist_t age actsmok drinking glu tg tc hdl/solution clparm;

run;

proc glm; where htnmed=0;

model sbp=wrist_t age actsmok drinking glu tg tc hdl/solution clparm;

run;

proc glm; where htnmed=0;

model sbp=whratio_t age actsmok drinking glu tg tc hdl/solution clparm;

run;

proc glm; where htnmed=0;

model sbp=whtratio_t age actsmok drinking glu tg tc hdl/solution clparm;

run;

proc glm; where htnmed=0;

model sbp=neck_t age actsmok drinking glu tg tc hdl/solution clparm;

run;

*++++++++++++++++++++++++++++++++++++++++++++++++++++++++++++++++++++++++++++++++++++++;

proc glm; where htnmed=0;

model dbp=bmi_t/solution clparm;

run;

proc glm; where htnmed=0;

model dbp=waist_t/solution clparm;

run;

proc glm; where htnmed=0;

model dbp=wrist_t/solution clparm;

run;

proc glm; where htnmed=0;

model dbp=whratio_t/solution clparm;

run;

proc glm; where htnmed=0;

model dbp=whtratio_t/solution clparm;

run;

proc glm; where htnmed=0;

model dbp=neck_t/solution clparm;

run;

proc glm; where htnmed=0;

model dbp=bmi_t age /solution clparm;

run;

proc glm; where htnmed=0;

model dbp=waist_t age /solution clparm;

run;

proc glm; where htnmed=0;

model dbp=wrist_t age /solution clparm;

run;

proc glm; where htnmed=0;

model dbp=whratio_t age /solution clparm;

run;

proc glm; where htnmed=0;

model dbp=whtratio_t age /solution clparm;

run;

proc glm; where htnmed=0;

model dbp=neck_t age /solution clparm;

run;

proc glm; where htnmed=0;

model dbp=bmi_t age actsmok drinking glu tg tc hdl/solution clparm;

run;

proc glm; where htnmed=0;

model dbp=waist_t age actsmok drinking glu tg tc hdl/solution clparm;

run;

proc glm; where htnmed=0;

model dbp=wrist_t age actsmok drinking glu tg tc hdl/solution clparm;

run;

proc glm; where htnmed=0;

model dbp=whratio_t age actsmok drinking glu tg tc hdl/solution clparm;

run;

proc glm; where htnmed=0;

model dbp=whtratio_t age actsmok drinking glu tg tc hdl/solution clparm;

run;

proc glm; where htnmed=0;

model dbp=neck_t age actsmok drinking glu tg tc hdl/solution clparm;

run;

*++++++++++++++++++++++++++++++++++++++++++++++++++++++++++++++++++++++++++;
